# Prior statin use and 90-day mortality in Gram-negative and Gram-positive bloodstream infection: a prospective observational study

**DOI:** 10.1007/s10096-014-2269-6

**Published:** 2014-11-06

**Authors:** A. Mehl, S. Harthug, S. Lydersen, J. Paulsen, B. O. Åsvold, E. Solligård, J. K. Damås, T.-H. Edna

**Affiliations:** 1 Department of Medicine, Levanger Hospital, Nord-Trøndelag Hospital Trust, Post Box 333, 7601 Levanger, Norway; 2 Unit for Applied Clinical Research, Department of Cancer Research and Molecular Medicine, Norwegian University of Science and Technology, Trondheim, Norway; 3 Department of Medicine, Haukeland University Hospital, Bergen, Norway; 4 Institute of Medicine, University of Bergen, Bergen, Norway; 5 Regional Centre for Child and Youth Mental Health and Child Welfare—Central Norway, Norwegian University of Science and Technology, Trondheim, Norway; 6 Centre of Molecular Inflammation Research, Department of Cancer Research and Molecular Medicine, Norwegian University of Science and Technology, Trondheim, Norway; 7 Department of Public Health, Norwegian University of Science and Technology, Trondheim, Norway; 8 Department of Endocrinology, St. Olav’s Hospital, Trondheim University Hospital, Trondheim, Norway; 9 Clinic of Anesthesia and Intensive Care, St. Olav’s Hospital, Trondheim University Hospital, Trondheim, Norway; 10 Department of Circulation and Medical Imaging, Norwegian University of Science and Technology, Trondheim, Norway; 11 Department of Infectious Diseases, St. Olav’s Hospital, Trondheim University Hospital, Trondheim, Norway; 12 Department of Surgery, Levanger Hospital, Nord-Trøndelag Hospital Trust, Levanger, Norway

## Abstract

In several studies on patients with bloodstream infection (BSI), prior use of statins has been associated with improved survival. Gram-positive and Gram-negative bacteria alert the innate immune system in different ways. We, therefore, studied whether the relation between prior statin use and 90-day total mortality differed between Gram-positive and Gram-negative BSI. We conducted a prospective observational cohort study of 1,408 adults with BSI admitted to Levanger Hospital between January 1, 2002, and December 31, 2011. Data on the use of statins and other medications at admission, comorbidities, functional status, treatment, and outcome were obtained from the patients’ hospital records. The relation of statin use with 90-day mortality differed between Gram-negative and Gram-positive BSI (*p*-value for interaction 0.01). Among patients with Gram-negative BSI, statin users had significantly lower 90-day total mortality [odds ratio (OR) 0.42, 95 % confidence interval (CI) 0.23–0.75, *p* = 0.003]. The association remained essentially unchanged after adjusting for the effect of sex, age, functional status before the infection, and underlying diseases that were considered confounders (adjusted OR 0.38, 95 % CI 0.20–0.72, *p* = 0.003). A similar analysis of patients with Gram-positive BSI showed no association of statin use with mortality (adjusted OR 1.22, 95 % CI 0.69–2.17, *p* = 0.49). The present study suggests that prior statin use is associated with a lower 90-day total mortality in Gram-negative BSI, but not in Gram-positive BSI.

## Background

In spite of antibiotic and supportive therapy, bloodstream infection (BSI) is still a major cause of mortality and morbidity [1–3]. Measures to improve outcomes from BSI are necessary. Several observational studies have assessed the relation between prior use of statins [3-hydroxy-3-methylglutaryl coenzyme A (HMG CoA) reductase inhibitors] and the outcome of BSI. The majority of the studies suggest that statin use could have a beneficial effect in patients with BSI [4–8], whereas some studies have shown no difference in mortality between statin users and non-users [9–11].

Many former studies assessing the relation between statin use and outcome of infection have not had verified BSI, nor have they discriminated between Gram-positive and Gram-negative etiology. As Gram-positive and Gram-negative bacteria alert the innate immune system in different ways [12, 13], drugs that have anti-inflammatory properties may not have the same effect in Gram-positive infection as they do in Gram-negative infection. Only a few authors have attempted to study this issue in patients [4, 7, 10, 11], and the results diverge. In this prospective observational cohort study on BSI, we chose to investigate the relation between prior statin use and 90-day mortality in Gram-positive and Gram-negative BSI separately.

## Materials and methods

Levanger Hospital serves a population of 87,000 as an emergency facility in a defined geographical area. Since 1994, all positive blood cultures at the hospital have been prospectively recorded for surveillance purposes, and clinical information has been recorded, in the following way: whenever a positive blood culture was reported, a physician at the clinical ward filled out a registration form. A team of three research nurses, a subordinate doctor, and the main investigator reviewed the patients’ records to verify the data and record additional variables. Data on statin use were prospectively included in the database from 2005, when we became aware of the early studies on statin use and sepsis. Information on the use of statins during the period 2002–2005 was recorded retrospectively from hospital records. The present study includes BSIs occurring between January 1, 2002 and December 31, 2011 in patients ≥16 years of age. BACTEC 9240 (Becton Dickinson Diagnostic Instrument Systems, Sparks, MD) was used for blood culture testing [14]. If a blood culture was positive for bacteria known to cause sepsis, only one positive vial was required for inclusion in the present study. For common skin contaminants (coagulase-negative staphylococci, alpha-hemolytic streptococci, corynebacteria, etc.), at least two positive vials from separate venipunctures were required. An episode of BSI was classified as polymicrobial if more than one organism was isolated from one or more blood cultures within a 72-h period.

For patients who had more than one episode with positive blood culture in the 10-year period, only the first episode was selected for this study. This cohort consisted of 1,408 patients. BSI with Gram-negative or Gram-positive bacteremia accounted for 1,356 episodes. The remaining cases consisted of eight candidemias and 44 with mixed polymicrobial infection.

The *exposure variable* was prior statin use, defined as taking a statin in the week before the time of positive blood culture [15]. Two patients, whose statins were discontinued more than one week before the date of the positive blood culture, were categorized as non-statin users. We recorded statin use and the specific statin and dosage from the patients’ hospital charts. The *outcome variable* was death from all causes within 90 days after the first positive blood culture [16]. The date of death was obtained from the patients’ electronic records, which is updated by the Civil Registration System in Norway. The following variables were *a priori determined as possible confounders* because they might be associated with both statin use and mortality from BSI (Table 1): age (<65 years, 65–79 years, ≥80 years); sex; comorbidities; Charlson comorbidity index [17], categorized as low (no underlying disease score), medium (score 1–2), or high (score >2) [7]; nursing home resident (yes/no); functional status (independent, partly independent, dependent, unknown); immunosuppressive therapy; alcohol abuse; smoking (no smoking, former smoker, present smoker); focus of infection (urinary tract, lungs, biliary tract, gastrointestinal tract, other, unknown); use of antibiotics before admission; and place of acquisition (community, healthcare, hospital). Whether the current BSI episode was nosocomial, healthcare-associated, or community-acquired was determined according to commonly used definitions [18, 19].

**Table 1:** Baseline characteristics of 1,356 adult patients with Gram-negative or Gram-positive bloodstream infection (BSI) by statin use at Levanger Hospital, Norway, 2002–2011

Variable	Gram-negative BSI (*n* = 784)			Gram-positive BSI (*n* = 572)		
	No statin use (*n* = 646)	Statin use (*n* = 138)	*p*-Value	No statin use (*n* = 474)	Statin use (*n* = 98)	*p*-Value
Age			<0.001			0.021
<65 years	185 (28.6)	25 (18.1)		159 (33.5)	26 (26.5)	
65–79 years	189 (29.3)	71 (51.4)		153 (32.3)	45 (45.9)	
≥80 years	272 (42.1)	42 (30.4)		162 (34.2)	27 (27.6)	
Female sex	367 (56.8)	67 (48.6)	0.060	196 (41.4)	35 (35.7)	0.29
Chronic renal insufficiency	52 (8.0)	16 (11.6)	0.17	36 (7.6)	19 (19.4)	<0.001
Malignancy						
Solid tumor	148 (22.9)	32 (23.2)	0.94	99 (20.9)	21 (21.4)	0.93
Hematological cancer	35 (5.4)	3 (2.2)	0.11	38 (8.0)	3 (3.1)	0.085
Diabetes mellitus	87 (13.5)	41 (29.7)	<0.001	70 (14.8)	35 (35.7)	<0.001
Hypertension	186 (28.8)	77 (55.8)	<0.001	127 (26.8)	40 (40.8)	0.003
Cardiovascular disease	212 (32.8)	91 (65.9)	<0.001	153 (32.3)	67 (68.4)	<0.001
Coronary heart disease	118 (18.3)	65 (47.1)	<0.001	88 (18.6)	50 (51.0)	<0.001
Congestive heart failure	59 (9.1)	16 (11.6)	0.36	49 (10.3)	14 (14.3)	0.25
Peripheral vascular disease	35 (5.4)	15 (10.9)	0.016	30 (6.3)	18 (18.4)	<0.001
Cerebral vascular disease	77 (11.9)	32 (23.2)	<0.001	52 (11.0)	18 (18.4)	0.041
Chronic liver disease	10 (1.5)	1 (0.7)	0.54	14 (3.0)	1 (1.0)	0.31
Chronic pulmonary disease	94 (14.6)	24 (17.4)	0.41	78 (16.5)	16 (16.3)	0.99
Rheumatological/immunological disease	55 (8.5)	13 (9.4)	0.72	44 (9.3)	11 (11.2)	0.56
Charlson comorbidity index			0.014			<0.001
Low (0)	187 (28.9)	23 (16.7)		144 (30.4)	10 (10.2)	
Medium (1–2)	278 (43.0)	69 (50.0)		209 (44.1)	43 (43.9)	
High (>2)	181 (28.0)	46 (33.3)		121 (25.5)	45 (45.9)	
Nursing home resident	85 (13.2)	8 (5.8)	0.021	47 (9.9)	2 (2.0)	0.017
Functional status prior to the present BSI			0.050*			0.035*
Independent	365 (56.5)	87 (63.0)		307 (64.8)	70 (71.4)	
Partly independent	178 (27.6)	38 (27.5)		108 (22.8)	26 (26.5)	
Dependent	97 (15.0)	11 (8.0)		52 (11.0)	2 (2.1)	
Unknown	6 (0.9)	2 (1.4)		7 (1.5)	0	
Immunosuppressive therapy	86 (13.3)	19 (13.8)	0.79	62 (13.1)	13 (13.3)	0.93
Alcohol abuse	30 (4.6)	4 (2.9)	0.45	20 (4.2)	2 (2.0)	0.38
Smoking						
Non-smoker	416 (64.4)	74 (53.6)	0.017	278 (58.6)	49 (50.0)	0.14
Former smoker	110 (17.0)	38 (27.5)	0.004	105 (22.2)	33 (33.7)	0.026
Present smoker	120 (18.6)	26 (18.8)	0.92	91 (19.2)	16 (16.3)	0.65
Focus of infection						
Urinary tract	358 (55.4)	76 (55.1)	0.98	45 (9.5)	10 (10.2)	0.83
Lungs	46 (7.1)	5 (3.6)	0.15	164 (34.6)	31 (31.6)	0.67
Biliary tract	95 (14.7)	26 (18.8)	0.21	14 (3.0)	5 (5.1)	0.32
Gastrointestinal tract	49 (7.6)	8 (5.8)	0.38	11 (2.3)	2 (2.0)	0.81
Skin or soft tissue	17 (2.6)	2 (1.4)	0.45	66 (13.9)	13 (13.3)	0.71
Other	25 (3.9)	4 (2.9)	0.69	105 (22.2)	28 (28.6)	0.16
Unknown	56 (8.7)	17 (12.3)	0.17	69 (14.6)	9 (9.2)	0.16
Systemic antibiotic therapy before admission	93 (14.4)	20 (14.5)	0.99	49 (10.3)	4 (4.1)	0.076
Place of acquisition						
Community-acquired	335 (51.9)	76 (55.1)	0.52	262 (55.3)	49 (50.0)	0.44
Acquired in hospital	76 (11.8)	21 (15.2)	0.28	73 (15.4)	16 (16.3)	0.81
Healthcare-associated	235 (36.4)	41 (29.7)	0.15	139 (29.3)	33 (33.7)	0.51
Variables expressing the severity of infection						
Severe sepsis or septic shock at the time of diagnosis	149 (23.1)	25 (18.1)	0.22	95 (20.0)	25 (25.5)	0.22
Severe organ failure (SOFA score >2 in any organ) at the time of diagnosis	90 (13.9)	19 (13.8)	>0.99	75 (15.8)	21 (21.4)	0.18
Stay in intensive care unit (ICU)	117 (18.1)	26 (18.8)	0.81	107 (22.6)	36 (36.7)	0.005
Appropriate initial antibiotic therapy	548 (84.8)	122 (88.4)	0.35	397 (83.8)	88 (89.8)	0.16

Data are presented as number of patients (%)

*Excluding unknowns

Variables expressing the *severity of the current BSI* (systemic inflammatory response syndrome, organ failure, hypotension, hypoperfusion, sepsis, severe sepsis, and septic shock [20]), severe organ failure (defined as SOFA score >2 in any organ [15, 21]), and admission to an intensive care unit (ICU) were recorded, but not considered confounders. Instead, they may be *mediators* in the pathway between prior statin use and mortality and, therefore, should not be adjusted for in the analyses [7, 22, 23].

Appropriate initial antibiotic therapy was defined as correctly dosed intravenous antibiotic therapy given within 24 h of the time that the blood culture specimen was obtained, with a regimen that was active in vitro against the microbe(s) isolated from blood culture(s) [24]. This variable was not adjusted for in the main analyses, as it was not considered a confounder. Appropriate initial antibiotic therapy is, indeed, strongly associated with outcome of BSI, but it is not associated with prior statin use in such a way that it influences whether a person has been prescribed statin medication. Prior statin use may be associated with the initial antibiotic therapy if statin use mitigates the inflammatory response so that symptoms are masked and, therefore, appropriate initial antibiotic therapy, is delayed. In this case, appropriate initial antibiotic therapy is a mediator in the pathway between statin use and death, and not a confounder. On the other hand, one might postulate some unknown variable that influences whether people are prescribed statin medication and also influences whether they receive appropriate initial antibiotic therapy (e.g., some underlying condition that is not included in the Charlson comorbidity index). To reduce the influence of such an unknown confounder, we also performed an analysis including adjustment for appropriate initial antibiotic therapy.

### Ethics

The Regional Committee for Ethics in Medical Research, Health Region IV, Norway approved the study. The Ethics Committee waived the need for informed consent because this was an observational study, the treatment of the patients was standard, and no samples were taken for the purposes of the research.

### Statistical analyses

Proportions were compared using the unconditional z-pooled test, which is the unconditional version of the Pearson Chi-squared test [25]. Unordered r × c tables were analyzed using the exact version of the Pearson Chi-squared test. The exact Cochran–Armitage test was used to test for trends in proportions. Univariable analysis of mortality curves was done with Kaplan–Meier analysis. The relation between prior statin use and 90-day total mortality was analyzed using logistic regression. Estimates were accompanied by 95 % confidence intervals (CIs). Two-sided *p*-values < 0.05 were considered significant. Statistical analyses were performed with SPSS 18 and StatXact 9.

## Results

During the 10-year period, 784 patients with Gram-negative BSI and 572 with Gram-positive BSI were identified. 17.6 % of those with Gram-negative and 17.1 % of those with Gram-positive BSI were statin users. The patient characteristics are shown in Table 1. In both Gram-negative and Gram-positive BSI, statin users had a higher burden of comorbid diseases, such as diabetes, hypertension, and cardiovascular disease, and they also had a higher Charlson comorbidity index. Variables expressing the severity of infection at the time of diagnosis were not significantly different in statin users and non-users. In Gram-positive BSI, the proportion of patients admitted to an ICU was higher in statin users.

The distribution of the microbial agents in relation to statin use is shown in Table 2. The distribution of microbes was essentially similar among statin users and non-users, except that *Klebsiella* spp. were more common among statin users and that some Gram-negative microbes (*Haemophilus influenzae*, *Neisseria meningitidis* and “Other aerobic Gram-negative organisms”) did not occur in statin users.

**Table 2:** Microbial agents in 784 episodes of Gram-negative BSI and 572 episodes of Gram-positive BSI, total and by statin use

Microbial agent(s)	No statin	Statin	Total	*p*-Value
Gram-negative microorganisms				
*Escherichia coli*	400 (61.9)	95 (68.8)	495 (63.1)	0.13
*Klebsiella* spp.	54 (8.4)	20 (14.5)	74 (9.4)	0.025
*Proteus* spp.	29 (4.5)	3 (2.2)	32 (4.1)	0.21
*Enterobacter* spp.	20 (3.1)	3 (2.2)	23 (2.9)	0.69
Other *Enterobacteriaceae*	19 (2.9)	6 (4.3)	25 (3.2)	0.44
*Pseudomonas* spp.	35 (5.4)	5 (3.6)	40 (5.1)	0.44
*Haemophilus influenzae*	15 (2.3)	0	15 (1.9)	0.079
*Neisseria meningitidis*	8 (1.2)	0	8 (1.0)	0.20
Other aerobic Gram-negative organisms	10 (1.5)	0	10 (1.3)	0.14
Anaerobic Gram-negative rods	28 (4.3)	5 (3.6)	33 (4.2)	0.88
Mixed Gram-negative aerobic or anaerobic rods	28 (4.3)	1 (0.7)	29 (3.7)	0.079
Total Gram-negative microorganisms	646 (100)	138 (100)	784 (100)	
Gram-positive microorganisms				
*Streptococcus pneumoniae*	162 (34.2)	27 (27.6)	189 (33.0)	0.23
*Staphylococcus aureus*	120 (25.3)	32 (32.7)	152 (26.6)	0.12
Beta-hemolytic streptococci	70 (14.8)	13 (13.3)	83 (14.5)	0.74
*Enterococcus* spp.	42 (8.9)	10 (10.2)	52 (9.1)	0.66
Viridans group streptococci	33 (7.0)	7 (7.1)	40 (7.0)	0.94
Coagulase-negative staphylococci	33 (7.0)	6 (6.1)	39 (6.8)	0.88
*Listeria monocytogenes*	3 (0.6)	1 (1.0)	4 (0.7)	0.88
Anaerobic Gram-positive microorganisms	6 (1.3)	1 (1.0)	7 (1.2)	0.89
Mixed Gram-positive aerobic or anaerobic BSI	5 (1.1)	1 (1.0)	6 (1.0)	0.68
Total Gram-positive microorganisms	474 (100)	98 (100)	572 (100)	

Data are presented as number of patients (%)

**Fig. 1:**
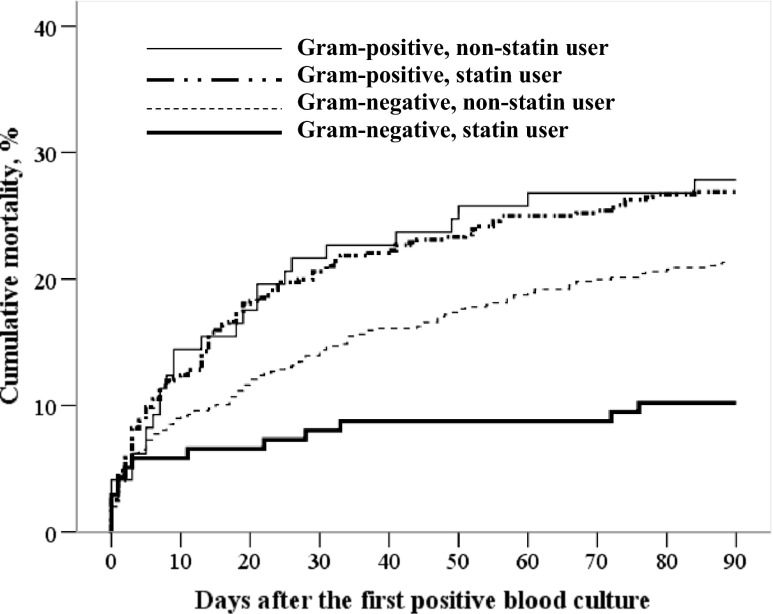
Mortality curves for patients with Gram-negative and Gram-positive bloodstream infection (BSI) stratified by statin use

Figure 1 shows the mortality curves for patients with Gram-negative and Gram-positive BSI. In Gram-negative BSI, the total 90-day mortality was 10.1 % (14/138) in statin users and 21.4 % (138/645) in non-statin users, *p* = 0.002. In Gram-positive BSI, the total 90-day mortality was 28.6 % (28/98) in statin users and 27.0 % (128/474) in non-statin users, *p* = 0.90.

The results of logistic regression analysis of the relation between statin use and 90-day total mortality are shown in Table 3. For the total BSI cohort, we found a negative association between prior statin use and 90-day mortality [odds ratio (OR) 0.69, 95 % CI 0.49–0.98, *p* = 0.040). The association was not mitigated by adjustment for possible confounding factors. Among patients with Gram-negative BSI, the mortality was significantly lower in statin users (OR 0.42, 95 % CI 0.23–0.75, *p* = 0.003). In Gram-positive BSI, there was no association between statin use and mortality. Adjustment for variables considered confounders did not weaken the association between statin use and 90-day mortality in Gram-negative BSI (adjusted OR 0.38, 95 % CI 0.20–0.72, *p* = 0.003). The relations between prior statin use and 90-day mortality in Gram-negative and Gram-positive BSI were significantly different; the adjusted OR for the interaction term was 2.95 (95 % CI 1.26–6.89, *p* = 0.012).

**Table 3:** Logistic regression analysis of the relation between prior statin use and 90-day total mortality

	No. of deaths/patients (%)	Unadjusted OR (95 % CI)	*p*-Value	Adjusted* OR (95 % CI)	*p*-Value
Any BSI					
No statin	285/1,164 (24.5)	1 (reference)		1 (reference)	
Statin	45/245 (18.4)	0.69 (0.49–0.98)	0.040	0.63 (0.43–0.95)	0.025
Gram-negative BSI					
No statin	138/646 (21.4)	1 (reference)		1 (reference)	
Statin	14/138 (10.1)	0.42 (0.23–0.75)	0.003	0.38 (0.20–0.72)	0.003
Gram-positive BSI					
No statin	128/474 (27.0)	1 (reference)		1 (reference)	
Statin	28/98 (28.6)	1.08 (0.67–1.75)	0.75	1.22 (0.69–2.17)	0.49

*Adjusted for age (<65 years, 65–79 years, ≥80 years); sex; Charlson comorbidity index; nursing home resident; functional status (independent, partly independent, dependent, unknown); immunosuppressive therapy; alcohol abuse; smoking (no smoking, former smoker, present smoker); focus of infection (urinary tract, lungs, biliary tract, gastrointestinal tract, other, unknown); use of antibiotics before admission; and place of acquisition (community, healthcare, hospital)

In an analysis including adjustment for appropriate initial antibiotic therapy, the effect estimates did not change (adjusted OR 0.38, 95 % CI 0.20–0.73, *p* = 0.004 and OR 1.21, 95 % CI 0.68–2.14, *p* = 0.52 for Gram-negative and Gram-positive BSI, respectively).

Adjusted ORs for the relation between statin use and 90-day mortality were similar when disease categories (e.g., diabetes, cardiovascular disease) were used instead of the Charlson comorbidity index for the total cohort (OR 0.62, 95 % CI 0.41–0.94, *p* = 0.026), Gram-negative BSI (OR 0.37, 95 % CI 0.18–0.74, *p* = 0.005), and Gram-positive BSI (OR 1.13, 95 % CI 0.62–2.07).

After excluding bacterial subgroups with zero statin users (*n* = 33), the association between statin use and 90-day mortality in Gram-negative BSI did not change, either unadjusted (OR 0.44, 95 % CI 0.24–0.78, *p* = 0.006) or after adjustment for confounders (OR 0.41, 95 % CI 0.21–0.78, *p* = 0.007).

For some variables included in the main analysis (focus of infection, use of antibiotics before admission, and place of acquisition), the association with prior statin use is unclear, and it is uncertain whether these variables fulfil the criteria for being a confounder. Additionally, we repeated the analysis after excluding these variables from the model, but the relations between prior statin use and 90-day mortality in Gram-negative (OR 0.37, 95 % CI 0.20–0.70, *p* = 0.002) and Gram-positive BSI (OR 1.00, 95 % CI 0.58–1.75) remained unchanged.

## Discussion

In this cohort study of patients with BSI, prior statin use was associated with reduced 90-day mortality in Gram-negative, but not in Gram-positive, BSI. To our best knowledge, no previous research has reported this finding. The effect measures were not changed by adjustment for variables that were considered confounding factors.

In the total cohort of BSI, the unadjusted OR for mortality among statin users compared with non-users did not differ much from what has been found by other investigators [7, 9, 11]. In one study, adjustment for possible confounding factors attenuated the effect measure to a value above 1 [11]. A population-based prospective cohort study on statin use and community-acquired pneumonia [26] raised concerns that previous studies indicating benefits of statins in patients with sepsis had been measuring and reporting a healthy user effect. In that study, statin users were less likely to die or to be admitted to an ICU. After adjusting for confounding factors that reflect patient frailty or healthy user behavior, no reduction in either mortality or the need for admission to an ICU in statin users was found.

In the present study, a significantly lower proportion of statin users were functionally dependent patients or nursing home residents compared with non-users. The proportion of former smokers was significantly higher among statin users. However, these associations of statin use were essentially similar in Gram-negative and Gram-positive BSI. Therefore, it seems unlikely that the observed difference in the possible preventive effect of statins between Gram-negative BSI and Gram-positive BSI in our study could be explained by a healthy user effect.

Most previous investigators have dealt with infection, sepsis, or BSI as if these categories represent homogenous groups with regard to pathogenesis or assumed preventive effect of statins. A few articles reported some data on the relations between statin use and death in Gram-positive and Gram-negative BSI separately. Liappis et al. [4] reported trends of reduced mortality rates in statin users with Gram-negative infection (1/19 vs. 48/223, *p* = 0.13) or *Staphylococcus aureus* infection (0/15 vs. 22/130, *p* = 0.13). Yang et al. [10] found a trend of lower mortality in statin users with Gram-negative bacteremia (10.3 % vs. 16.4 %, *p* = 0.25), but not in Gram-positive bacteremia (33.3 % vs. 27.8 %, *p* = 0.50). Thomsen et al. [7] did not find such a trend in the enterobacterial group of Gram-negative bacteria (adjusted 30-day mortality rate ratio 1.07, 95 % CI 0.61–1.86, *p* = 0.82), nor did Leung et al. find a negative association between statin use and death in Gram-negative BSI (adjusted hazard ratio 1.10, 95 % CI 0.87–1.39) [11]. However, the latter had adjusted for variables expressing the severity of the current infection, i.e., factors that should be considered as mediators rather than confounders, and such an adjustment may attenuate the true associations [22]. Recently, López-Cortés et al. found a negative association between statin use and death within 14 days from *Staphylococcus aureus* BSI (adjusted OR 0.08, 95 % CI 0.01–0.66, *p* = 0.02) [8].

Statins are renowned for their anti-inflammatory and pleiotropic lipid-lowering independent effects. They exert their anti-inflammatory effects through their implication on a variety of molecular pathways of the innate and adaptive immune system, such as their impact on the circulating levels of inflammatory cytokines, as well as anti-coagulant and anti-proliferative effects [27, 28]. With regards to sepsis, many in vitro and animal studies have demonstrated that statins attenuate endotoxin-induced inflammatory responses [29]. Similarly, statins have been found to diminish inflammatory responses induced by *Staphylococcus aureus* lipoteichoic acid [30]. However, there are few studies that have explored the mechanisms for differential effects of statins in Gram-positive and Gram-negative BSI, as observed in the present study. Gram-positive and Gram-negative bacteria have different cell wall components and may stimulate different toll-like receptors (TLRs) on cells of the innate immune system, initiating the transcription of different inflammatory mediator genes. Interestingly, there are several studies showing that statins decrease lipopolysaccharide (LPS) signaling through the downregulation of TLR4 expression on monocytes [31]. Although some studies suggest that statins may also suppress TLR2 signaling in Gram-positive infection, statins may not inhibit the lipoprotein-induced pathway via TLR2 and the LPS-induced pathway via TLR4 to the same extent [32]. Indeed, there are some studies showing that statins inhibit TLR4-mediated activation of interferon regulatory factor 3 (IRF3) and interferon-beta production in macrophages stimulated by LPS, while no such effects were observed for TLR2 stimulation [33]. The clinical relevance of these findings is, however, unknown and require further examination. Nevertheless, they illustrate that there may be molecular mechanisms supporting the findings of different effects of statins in Gram-positive and Gram-negative BSI.

Our findings indicate that the possible preventive effect of statins in BSI may be a subgroup effect seen in Gram-negative, but not in Gram-positive, infection. Whether a negative association between statin use and mortality in Gram-negative BSI really exists as a biological interaction should be further investigated, preferably by randomized controlled studies (RCTs). As of yet, few RCTs have been performed [34, 35], and though several are presently in progress [36], they are designed to study the effect of statin treatment in ongoing infection, not the prophylactic effect of statin use prior to infections. One RCT has shown improved survival in atorvastatin-treated ICU patients with severe sepsis if they had been prior statin users, but no effect was found in patients who received de novo statin therapy [35]. RCTs, so far, have not assessed whether different etiologic agents influence the relation between prior statin use and the outcome of infections.

### Strengths and weaknesses of the study

The strength of our study is that our bacteremia database contains confirmed diagnoses and variables collected from the patients’ hospital data, which include medical records. Thus, we have more reliable data than diagnostic information from only discharge databases. Our cohort has a sufficient number of patients in the different groups, which makes it possible to assess the relation between statin use and 90-day mortality, an important endpoint in prior studies.

The study has the weakness of being an observational study, burdened with the risk of confounding. Socioeconomic factors have not been adjusted for, but severe confounding by socioeconomic differences seems unlikely because statin prescriptions are reimbursed, and access to hospital treatment is the same for all citizens in Norway. In addition, we have not adjusted for vaccinations, e.g., pneumococcus vaccine or influenza vaccine, which some previous authors have regarded as “healthy user” markers. Nonetheless, we are not aware of confounders that would be likely to cause a strong association of statin use with reduced mortality in Gram-negative, but not in Gram-positive, BSI.

## Conclusion

In the present study, the relation between statin use and 90-day total mortality was different in Gram-positive and Gram-negative bloodstream infection (BSI). In Gram-negative BSI, statin use was associated with lower 90-day mortality, which was not the case in Gram-positive BSI.
